# Supplementary material for: Triplet-driven chemical reactivity of β-carotene and its biological implications
Source: Nat Commun. 2022 May 5;13:2474. doi: 10.1038/s41467-022-30095-z (PMC9072317; doi:10.1038/s41467-022-30095-z)
Supplement: Supplementary file 1 — Supplementary Information [file 41467_2022_30095_MOESM1_ESM.pdf]

Supplementary Information

Triplet-driven chemical reactivity of β-carotene and its biological implications

Mateusz Zbyradowski[1], Mariusz Duda[1], Anna Wisniewska-Becker[1], Heriyanto[1,2], Weronika Rajwa[1], Joanna Fiedor[3], Dragan Cvetkovic[4], Mariusz Pilch[1,5] , and Leszek Fiedor*[1]

[1]Faculty of Biochemistry, Biophysics and Biotechnology, Jagiellonian University, Gronostajowa 7, 30-387 Cracow, Poland

[2]Ma Chung Research Center for Photosynthetic Pigments, Ma Chung University Villa Puncak Tidar N-01, Malang 65151, Indonesia

[3]Faculty of Physics and Applied Computer Science, AGH-University of Science and Technology, 30-059 Cracow, Mickiewicza 30, Poland

[4]Faculty of Technology, University of Niš, 16000 Leskovac, Serbia

[5]Faculty of Chemistry, Jagiellonian University, Gronostajowa 2, 30-387 Kraków

*Corresponding author, Email: leszek.fiedor@uj.edu.pl

Table of Contents

Suppl. Figs 1 – 4 The breakdown of β-carotene endoperoxides in the dark monitored using mass spectrometry and electronic absorption spectroscopy.

Suppl. Fig. 5 RP-HPLC analysis with in-line diode-array recording of electronic absorption spectra of the purified β-carotene (substrate) and its oxygenation products.

Suppl. Fig. 6 The DFT-predicted vacuum-relaxed conformations of β-carotene, its two endoperoxides, βCar-5,8-EPO and βCar-7,10-EPO, and the corresponding isomeric carotenes formed after the release of the O_2 moiety.

Suppl. Fig. 7 The electronic absorption spectrum of β-carotene recorded in acetone and the expanded (~100×) region between 600 and 750 nm showing the non-zero activity of the S_2 transition (inset).

Suppl. Fig. 8 Changes in the emission lifetime of $^1O_2$ in the presence of βCar or βCar-5,8-EPO in acetone.

Suppl. Fig. 9 The DMPO-spin trapping of free radicals generated by βCar-5,8-EPO in liposomes.

Suppl. Fig. 10 The positioning of β-carotenes in Photosystem II and xanthophylls in LHCII.

Suppl. Table 1 The DFT-computed total energies of β-carotene (βCar), the O_2 molecule and other compounds involved in the formation and breakdown of β-carotene endoperoxides.

Suppl. Table 2 The DFT-computed total energies and conformations of the endoperoxide moieties of βCar-5,8-EPO and βCar-7,10-EPO truncated at various sites.

Suppl. Table 3 Comparison of the ab initio computed solvent effects on the basic electronic properties of βCar, βCar-5,8-EPO and βCar-7,10-EPO.

Breakdown of β-carotene-5,8-endoperoxide

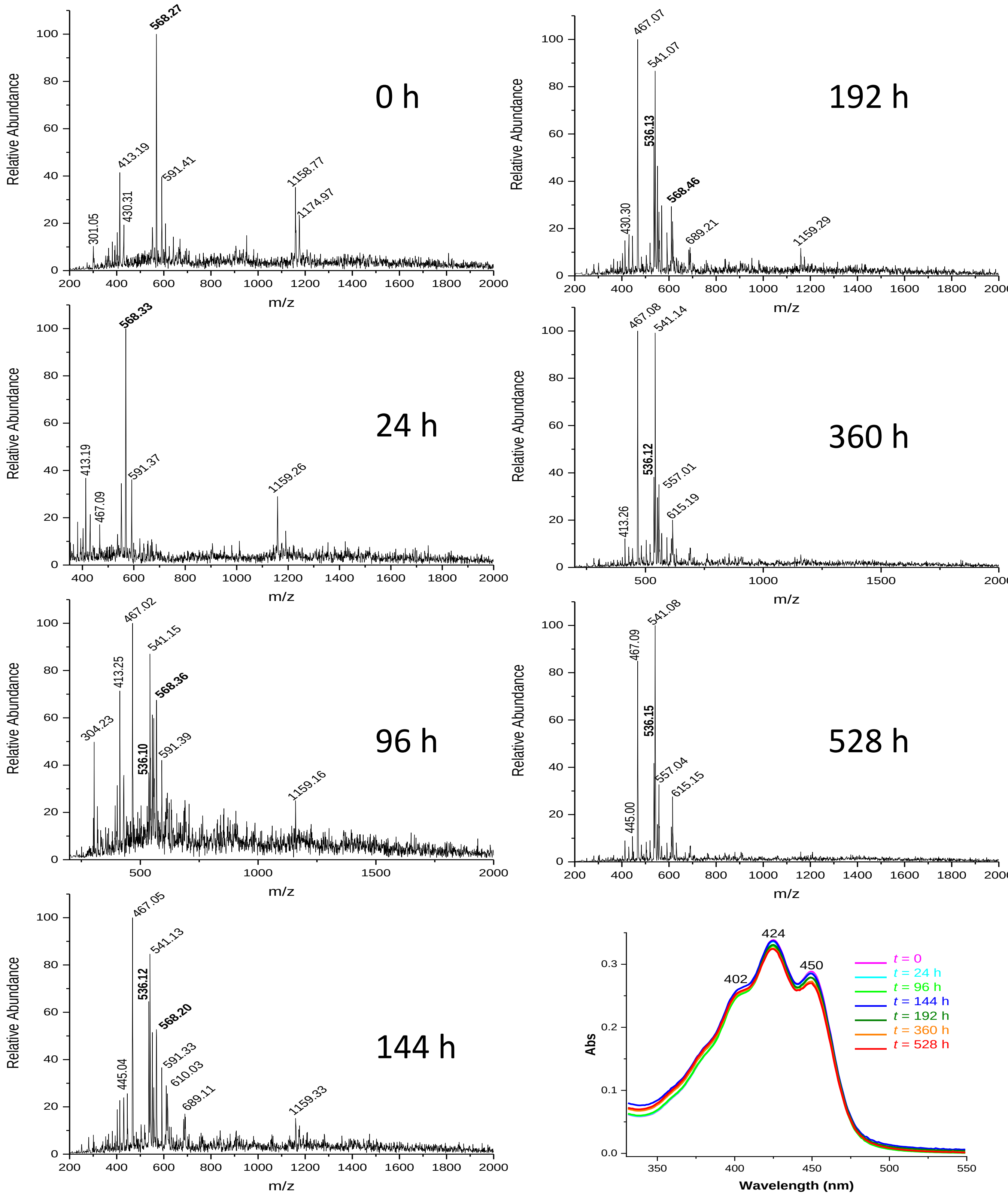

Supplementary Fig. 1 The breakdown of β-carotene-5,8-endoperoxide in methanol at ambient temperature in the dark monitored using mass spectrometry and electronic absorption spectroscopy. The experimental details are given under the Methods section.

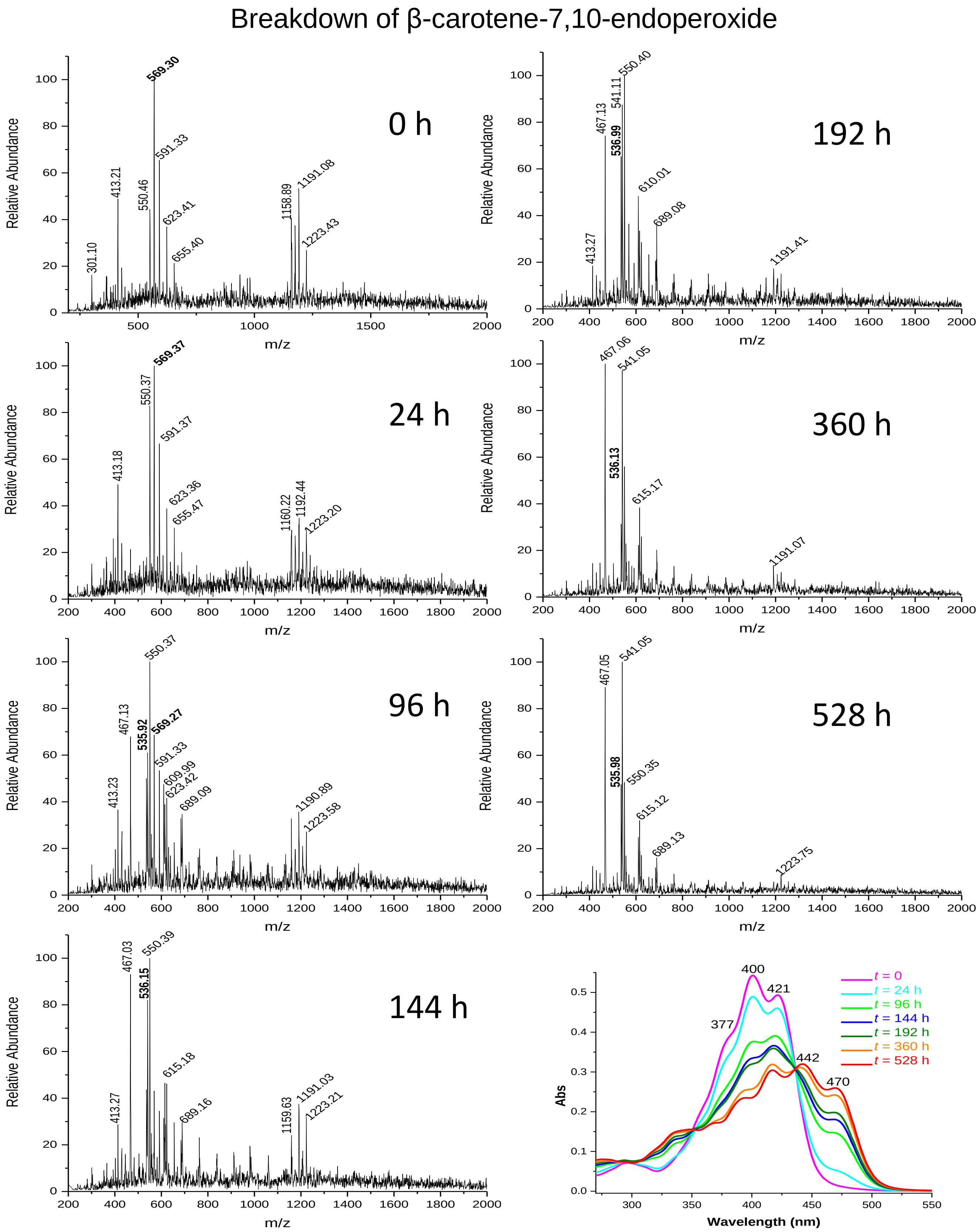

Supplementary Fig. 2 The breakdown of β-carotene-7,10-endoperoxide in methanol at ambient temperature in the dark monitored using mass spectrometry and electronic absorption spectroscopy. The experimental details are given under the Methods section.

Breakdown of β-carotene-5,8,7',10'-diendoperoxide

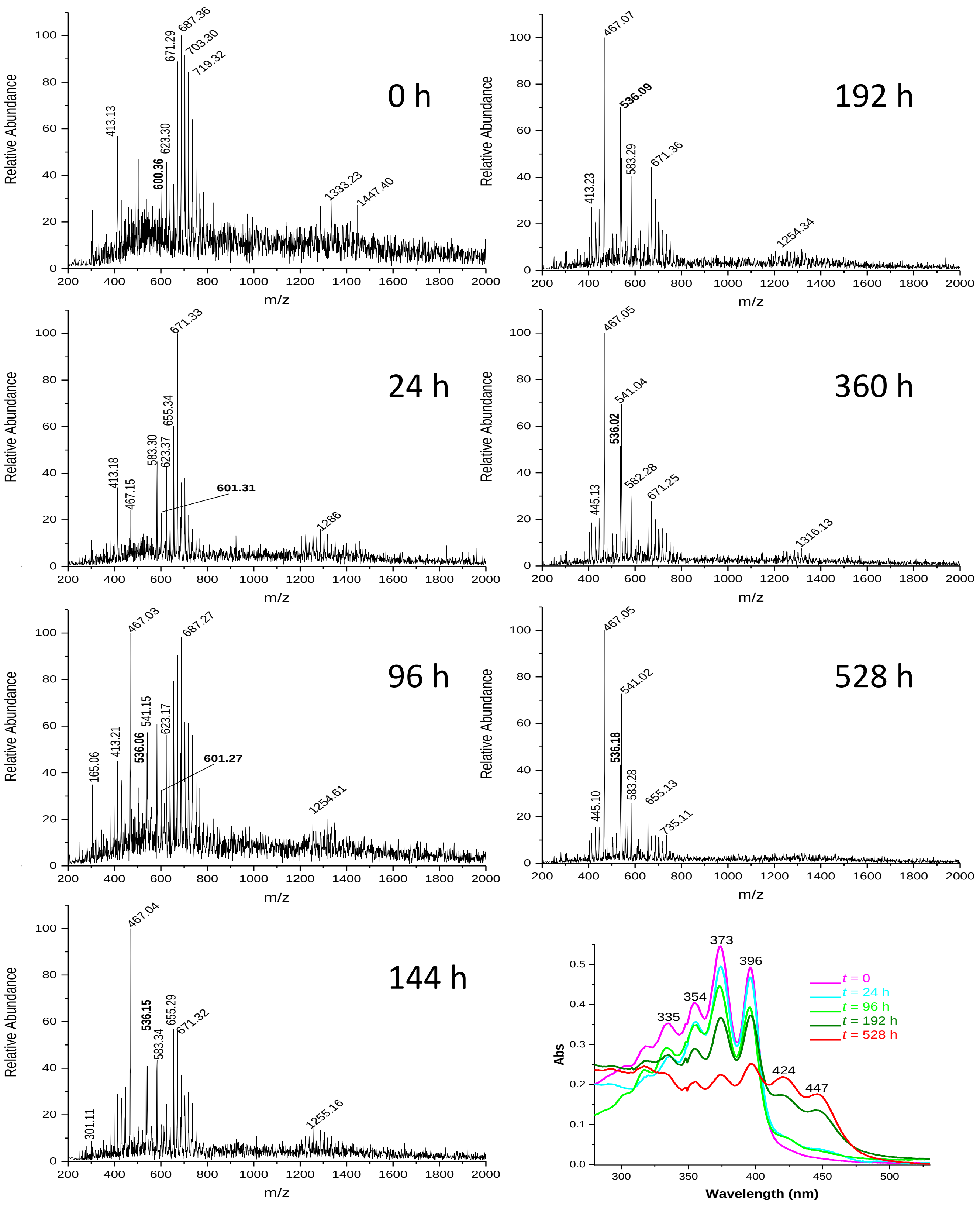

Supplementary Fig. 3 The breakdown of β-carotene-5,8,7',10'-diendoperoxide in methanol at ambient temperature in the dark monitored using mass spectrometry and electronic absorption spectroscopy. The experimental details are given under the Methods section.

Breakdown of β-carotene-5,8,5',8'-diendoperoxide

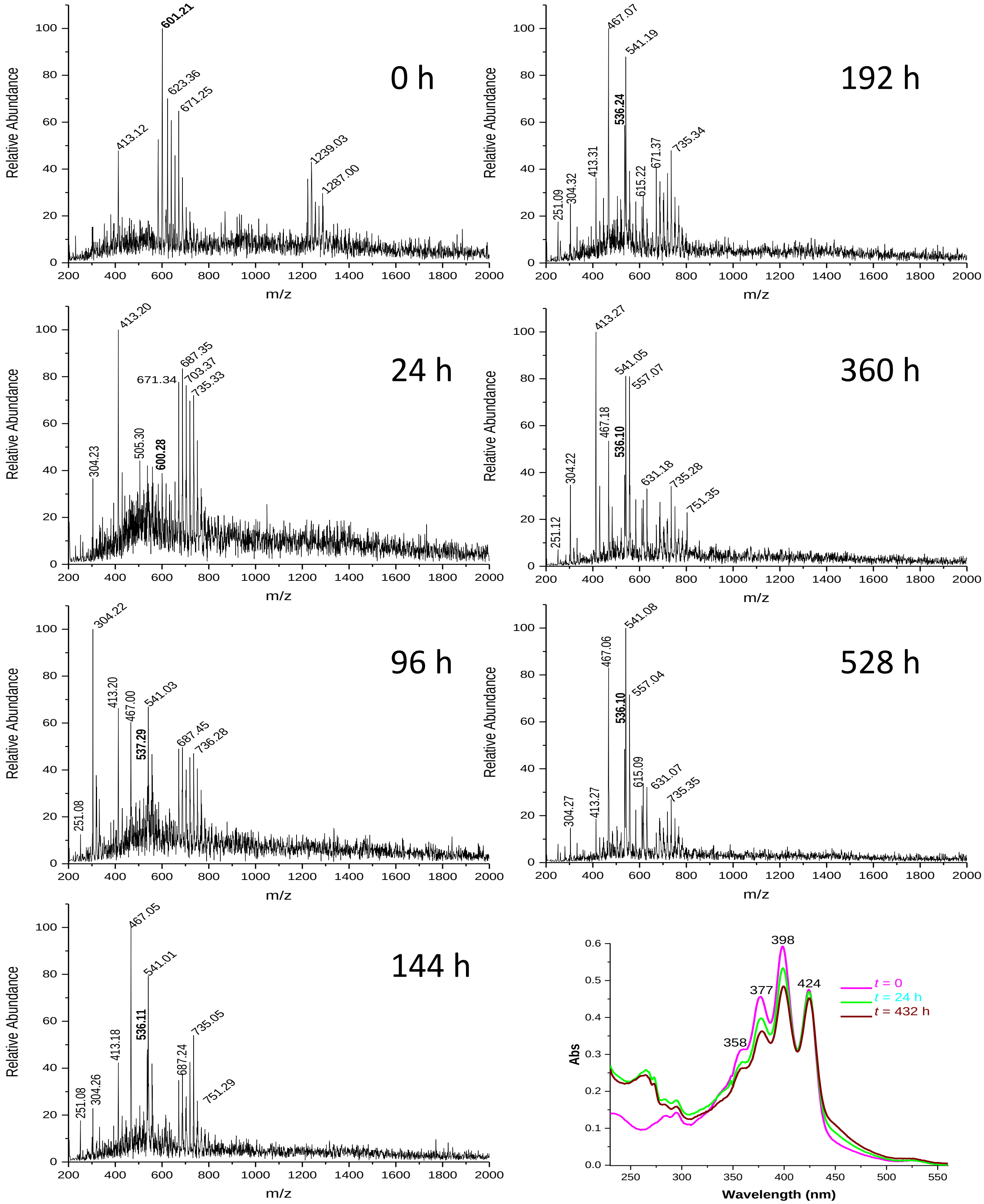

Supplementary Fig. 4 The breakdown of β-carotene-5,8,5',8'-diendoperoxide in methanol at ambient temperature in the dark monitored using mass spectrometry and electronic absorption spectroscopy. The experimental details are given under the Methods section.

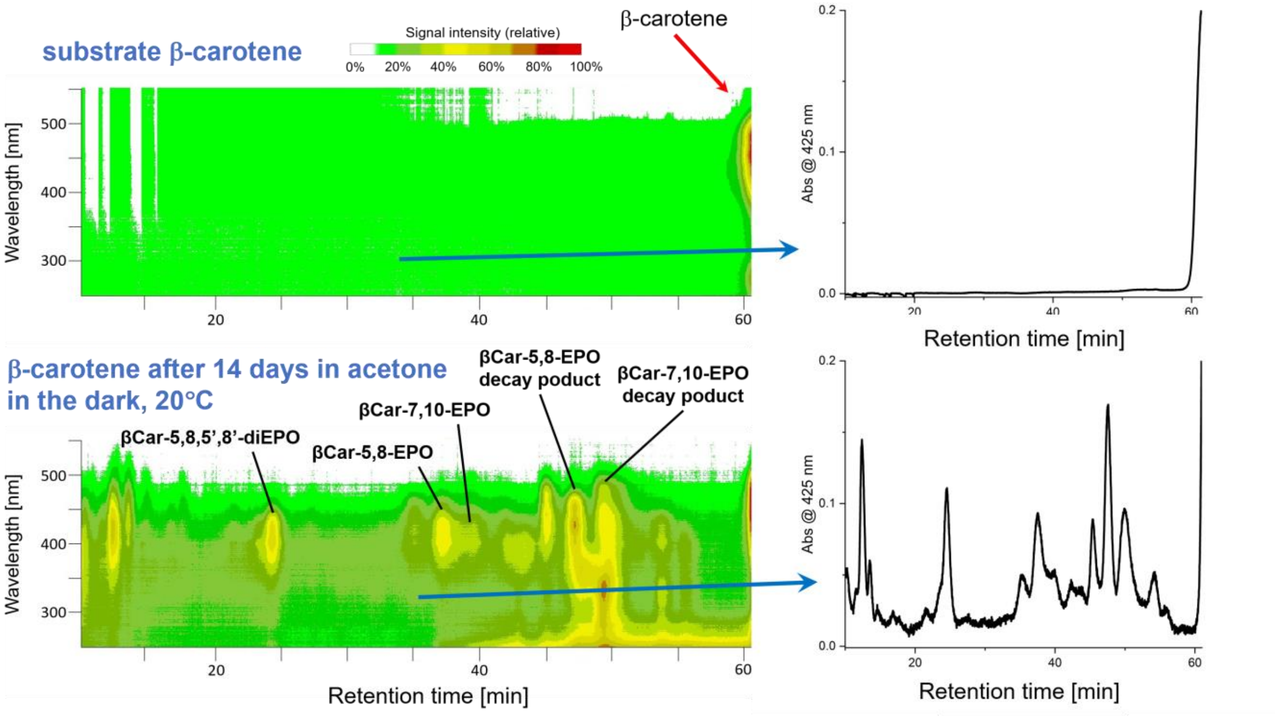

Supplementary Fig. 5 Upper panel: the RP-HPLC analysis with in-line diode-array recording of electronic absorption spectra of the purified β-carotene used as the substrate in the oxygenation reactions. On the right, the corresponding chromatogram recorded at 425 nm. Lower panel: the RP-HPLC analysis with in-line diode-array recording of electronic absorption spectra of the products of β-carotene oxygenation in acetone obtained after two weeks of standing at room temperature in the dark, showing the presence of βCar-EPOs and their decay products. On the right, the corresponding chromatogram recorded at 425 nm. The conditions of the separation are given in the Materials and Methods section.

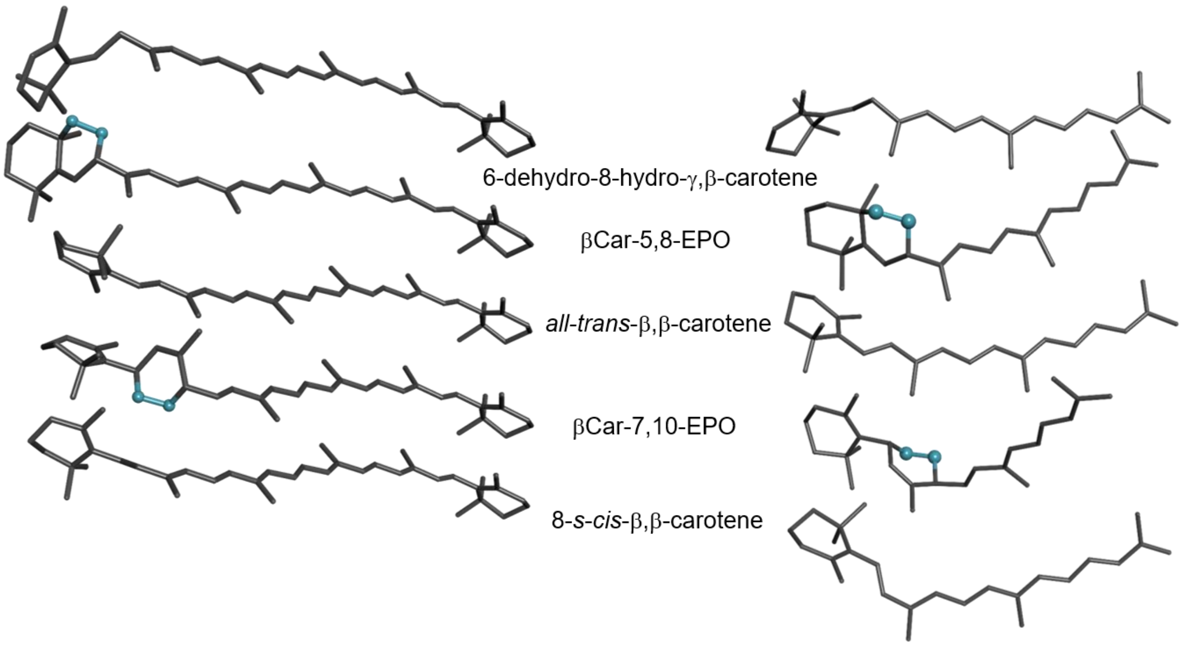

Supplementary Fig. 6 The DFT-predicted vacuum-relaxed conformations of β-carotene (middle), its two endoperoxides, βCar-5,8-EPO and βCar-7,10-EPO, and the corresponding isomeric carotenes formed after the release of the O_2 moiety. Left: entire molecules; right: the expanded and rotated fragments which suffer deformation due to the addition/release of O_2 moiety.

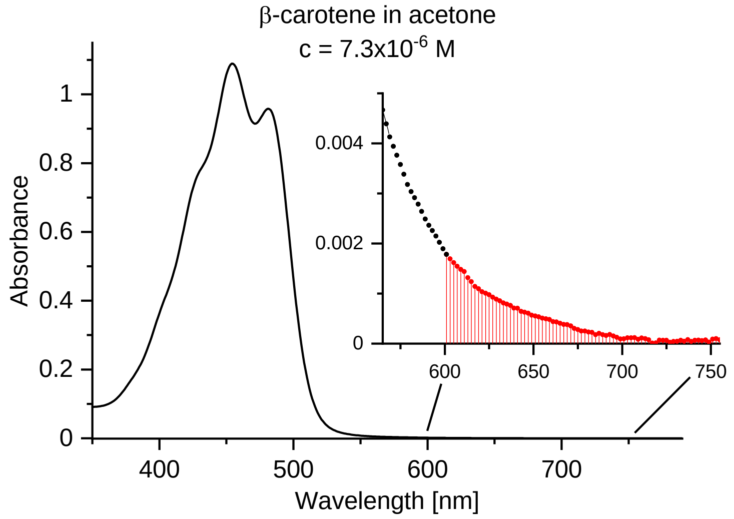

Supplementary Fig. 7 The electronic absorption spectrum of β-carotene recorded in acetone and the expanded (~100×) region between 600 and 750 nm showing the non-zero activity of the S_2 transition (inset).

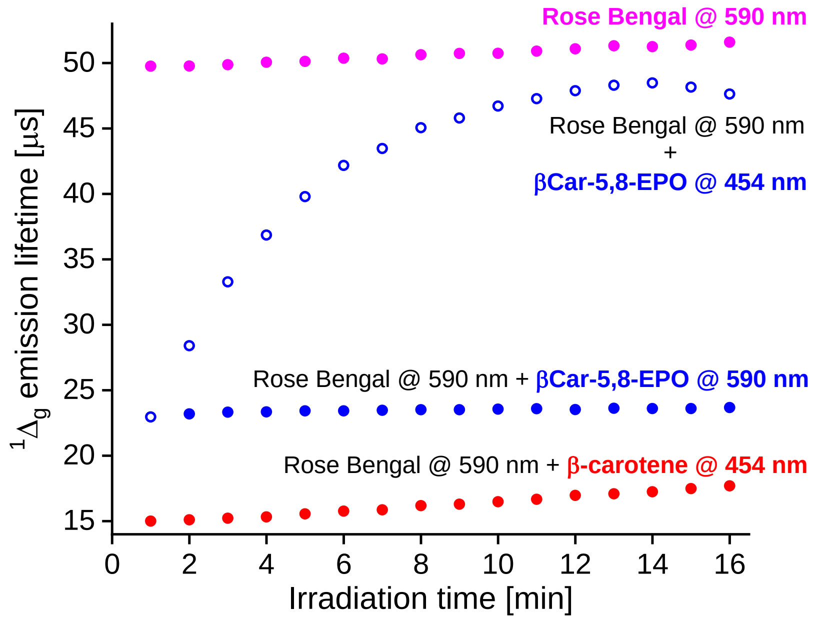

Supplementary Fig. 8 Changes in the emission lifetime of $^1O_2$ generated by Rose Bengal (2 μM) alone (rose dots) and in its mixtures with either βCar or βCar-5,8-EPO in acetone, using 590 nm excitation. βCar (red dots) and βCar-5,8-EPO (empty blue dots), each at 2.66 μM, were independently irradiated at 454 nm with laser flashes (1 kHz). Note a large increase in the $^1O_2$ lifetime in the mixture of Rose Bengal and βCar-5,8-EPO irradiated at 454 nm, and practically no effect of the irradiation at 590 nm.

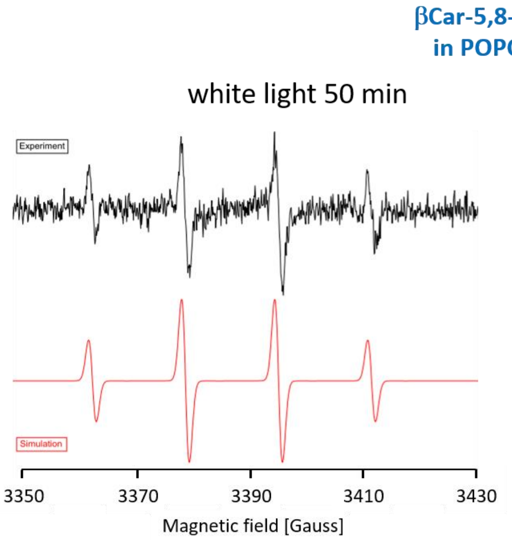

Supplementary Fig. 9 The DMPO-spin trapping of free radicals generated by βCar-5,8-EPO (35 μM) in the 1-palmitoyl-2-oleoyl-*sn*-glycero-3-phosphocholine (POPC) multilamellar liposomes under white light (30 mW/cm^2). The EPR spectra were recorded in the presence of 80 μM DMPO during 25 min (left) and 70 min (right) of illumination. Upper traces – experimental spectra; lower traces – spectra simulated by WinSim program using parameters: a_N = 14.95, a_H = 14.92 G. No DMPO-OH adducts were detected in the liposomes alone neither for βCar-5,8-EPO in liposomes in the dark. Source data are provided as a Source Data file.

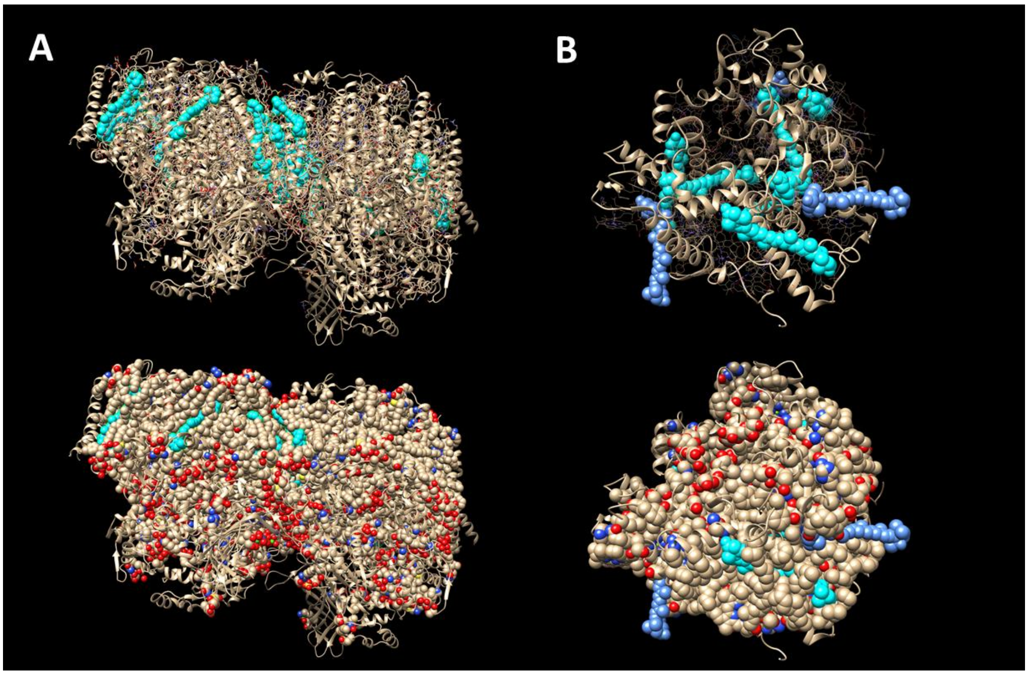

Supplementary Fig. 10 The positioning of (**A**) β-carotenes (in cyan) in Photosystem II (PDB entry 3WU2) and (**B**) luteins (in cyan) and neoxanthins (in blue) in LHCII (PDB entry 2BHW). In the lower panels, the atomic van der Waals radii are shown to indicate a very dense packing of the complexes, which prevents penetration of the O_2 molecules in the protein interior. β-Carotenes and luteins are well buried within the complexes and are protected from the O_2 attack. Neoxanthins are exposed, having their 6-8 allenic and 3,5-dihydroxy moieties protruding into the lipid bilayer. The images were prepared using a Chimera 1.16 software.

Supplementary Table 1

The DFT-computed total energies of β-carotene (βCar), the O_2 molecule and other compounds involved in the formation and breakdown of β-carotene endoperoxides. The structures of the molecules used in the computations are also shown. For βCar and its derivatives, the predicted and experimental energies of the first electronic transitions (in vacuo) are listed. The structure numbering as in Fig. 4 in the main text.

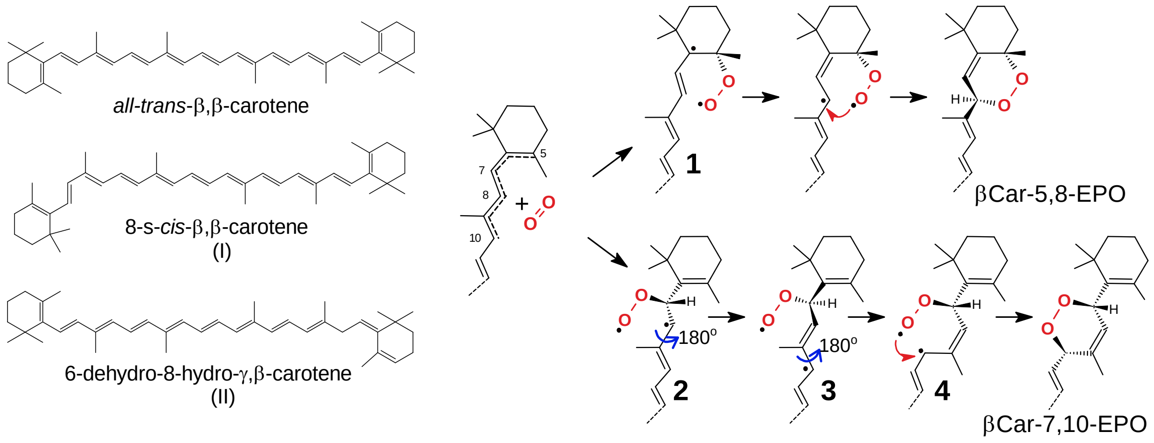

	DFT total energy	stabilization energy (difference to parental system)		energy of the 1st allowed electronic transition (S_2)	
	[hartree]	[hartree]	[kJ/mol]	predicted [cm^{-1} (nm)]	experimental (0-1 band) [cm^{-1} (nm)]
βCar	- 1557.9089	0	0	17986 (556)	22026 (454)
"half-flat" βCar*	- 1557.8976	- 0.0113	- 29.67		
carotene I	- 1557.8966	- 0.0123	- 32.29	18051 (554)	23310 (429)
carotene II	- 1557.8877	- 0.0212	- 55.66	19608 (510)	25707 (389)
O_2	-150.2574				
βCar + O_2	-1708.1663	0	0		
βCar-5,8-EPO	-1708.2559	+ 0.0896	+ 235.24	19608 (510)	23474 (426)
βCar-7,10-EPO	-1708.2392	+ 0.0729	+ 191.40	20661 (484)	24876 (402)
O_2 attack on C8	-1708.1787	+ 0.0124	+ 32.56		
O_2 attack on C5 (**1**)	-1708.2052	+ 0.0389	+ 102.13		
O_2 attack on C10	-1708.1840	+ 0.0177	+ 46.47		
O_2 attack on C7 (**2**)	-1708.2034	+ 0.0371	+ 97.41		
C8-C9 rotation product (**3**)	-1708.1985	+ 0.0322	+ 84.54		
C9-C10 rotation product (**4**)	-1708.1928	+ 0.0265	+ 69.58		

*one ionone ring forced into the molecular plane

Supplementary Table 2

The DFT-computed total energies and conformations of the endoperoxide moieties of βCar-5,8-EPO and βCar-7,10-EPO truncated at various sites of the molecules, as indicated in the structural formulas. The predicted conformations (in vacuo) of the truncated molecules (color-coded) are overlapped at the -O-O- bridges for a better comparison.

truncated βCar-5,8-EPO	total energy [hartree]	predicted conformation (overlapped)	truncated βCar-7,10-EPO	total energy [hartree]	predicted conformation (overlapped)
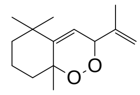 (green)	- 897.0976		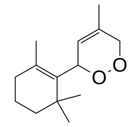 (green)	- 897.0947	
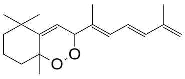 (red)	- 891.2328	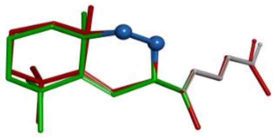	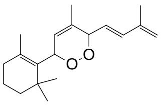 (red)	- 891.2167	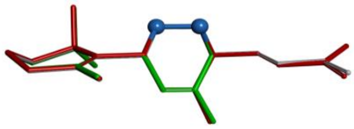
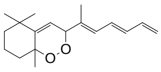 (gray)	- 851.9126		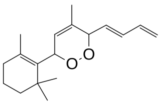 (gray)	- 851.8959	

Supplementary Table 3

Comparison of the solvent effects on the basic electronic properties of *all-trans*-β,β-carotene (βCar), βCar-5,8-EPO and βCar-7,10-EPO, computed with the use of the TD-DFT method within the PCM model.

carotenoid	medium	Ground state energy [hartree]	ΔE (pairwise) [kJ/mol]	Dipole Moment [D]	Polarizability [Bohr^3]	HOMO energy [hartree]	LUMO energy [hartree]	ΔLH [cm^{-1}/nm]	1st allowed electronic transition (S_2) [nm]	oscillator strength
βCar	cyclohexane	-1557.9043		0.136	1106.92	-0.16257	-0.08107	17886.93 / 559.1	592	3.925
	acetone	-1557.9090		0.138	1308.94	-0.16511	-0.08387	17829.87 / 560.9	594	3.927
	ethanol	-1557.9094		0.153	1314.46	-0.16519	-0.08396	17827.67 / 560.9	594	3.927
	H_2O	-1557.9096		0.153	1332.95	-0.16546	-0.08425	17823.28 / 561.1	594	3.928
βCar-5,8-EPO	cyclohexane	-1708.2600	–	2.647	941.74	-0.16576	-0.07781	19302.52 / 518.1	548	3.512
	acetone	-1708.2668	–	3.189	1105.27	-0.16876	-0.08103	19254.24 / 519.4	545	3.493
	ethanol	-1708.2670	–	3.205	1109.78	-0.16885	-0.08113	19252.04 / 519.4	545	3.493
	H_2O	-1708.2676	–	3.261	1124.81	-0.16917	-0.08147	19247.65 / 519.5	544	3.489
βCar-7,10-EPO	cyclohexane	-1708.2428	+45.16	2.912	828.42	-0.17041	-0.07631	20652.27 / 484.2	509	3.067
	acetone	-1708.2488	+47.26	3.872	970.93	-0.17319	-0.07983	20489.86 / 488.0	510	3.022
	ethanol	-1708.2490	+47.26	3.902	974.99	-0.17328	-0.07995	20483.28 / 488.2	511	3.021
	H_2O	-1708.2498	+46.73	4.872	998.25	-0.17437	-0.08191	20292.34 / 492.8	515	2.974
